# Supplementary material for: Content-rich biological network constructed by mining PubMed abstracts
Source: BMC Bioinformatics. 2004 Oct 8;5:147. doi: 10.1186/1471-2105-5-147 (PMC528731; doi:10.1186/1471-2105-5-147)
Supplement: Additional File 2 — The original results of the above study (non-essential files are deleted to keep the file size under the limit set by BMC bioinformatics). [file 1471-2105-5-147-S2.bz2 › chilibotAdditionalFile2/dip10/26ID8999548E89/html/IFNG_IL18.html]

 


 **IFNG** and **IL18** 
  
Found 772 abstracts in PubMed, retrieved 10.  
 

 What does Google say? 
 PDF only 
| .edu only 

---

**Interactive relationship** (e.g. stimulation, inhibition, etc)

**Neutral relationship**- In the gut of non infected piglets, IL 1 beta, IL 8 and IL 18  [ **IL18** ]  were detected whereas TNF alpha and IFN gamma  [ **IFNG** ]  were mostly missing.  Ref: 12879755 Folia Microbiol (Praha), 2003
- CD4 and CD8 T cells induced IL 18  [ **IL18** ]  from DC and primed for IL 12 p70 secretion via interferon gamma  [ **IFNG** ]  IFN gamma  [ **IFNG** ]  or tumour necrosis factor alpha TNF alpha.  Ref: 12871213 Immunology, 2003
- Interleukin 18  [ **IL18** ]  IL 18  [ **IL18** ] , a monokine that shares functional abilities with IL 12, is a potent inductor of interferon gamma  [ **IFNG** ]  IFN gamma  [ **IFNG** ]  by Th1 and natural killer NK cells.  Ref: 12858406 J Med Virol, 2003
- The protective effect of IL 18  [ **IL18** ]  bp Fc was accompanied by modified ex vivo immune responses, in that spleen cells and peritoneal macrophages contained fewer IFN gamma  [ **IFNG** ]  secreting cells and released lower amounts of nitrite an index of nitric oxide production and IL 1beta.  Ref: 12884303 Eur J ImmunolEur J Immunol, 2003
- IL 18  [ **IL18** ]  is a cytokine structurally and functionally related to IL 1 that, in synergy with IL 12, stimulates the synthesis of IFN gamma  [ **IFNG** ]  from T lymphocytes and natural killer cells.  Ref: 12884303 Eur J ImmunolEur J Immunol, 2003

**Non-interactive relationship** (e.g. studied together, co-existance, homology, etc.)

- However, mRNA expression for IL 18  [ **IL18** ]  and interferon gamma  [ **IFNG** ]  IFN gamma  [ **IFNG** ]  was downregulated, and the expression of mRNA for the anti inflammatory cytokine transforming growth factor beta4 TGF beta 4 was upregulated.  Ref: 12859858 J Interferon Cytokine Res, 2003
- Sepsis is initiated and perpetuated by the overzealous systemic production of proinflammatory cytokines such as tumor necrosis factor TNF alpha, interferon IFN gamma  [ **IFNG** ] , interleukin IL 12, and IL 18  [ **IL18** ]  sometimes resulting in excessive tissue injury and death.  Ref: 12874588 Surgery, 2003
- Furthermore, serum IL 12 and IL 18  [ **IL18** ]  levels and liver MNCs IL 12, IL 18  [ **IL18** ] , and IFN gamma  [ **IFNG** ]  production were significantly increased in CLP mice compared with sham mice after LPS challenge.  Ref: 12874588 Surgery, 2003
- Recent reports demonstrated that P. acnes treatment promotes IL 12 and IL 18  [ **IL18** ]  synthesis in mice inducing IFN gamma  [ **IFNG** ]  release, enhancement of IgG2a switch and inhibition of Th2 cell expansion.  Ref: 12880687 Immunol Lett, 2003
- Because IFN gamma  [ **IFNG** ]  plays a key pathogenic role in the development of murine immunoinflammatory diabetes induced by multiple low doses of streptozotocin STZ we investigated the effect of negating the actions of endogenous IL 18  [ **IL18** ]  in this model by administering recombinant IL 18  [ **IL18** ]  binding protein Fc IL 18  [ **IL18** ]  bp Fc.  Ref: 12884303 Eur J ImmunolEur J Immunol, 2003
- uNK cells proliferate, produce cytokines interferon gamma  [ **IFNG** ]  IFN gamma  [ **IFNG** ]  and interleukin 18  [ **IL18** ]  IL 18  [ **IL18** ]  and IL 27, and terminally differentiate into granulated lymphocytes.  Ref: 12887272 Reproduction, 2003
- IL 12, IL 18  [ **IL18** ] , and IFN gamma  [ **IFNG** ]  levels in supernatants of the liver MNCs stimulated with LPS in CLP mice were significantly higher than those in sham mice 7 days after the procedure.  Ref: 12874588 Surgery, 2003
